# Supplementary material for: Content-rich biological network constructed by mining PubMed abstracts
Source: BMC Bioinformatics. 2004 Oct 8;5:147. doi: 10.1186/1471-2105-5-147 (PMC528731; doi:10.1186/1471-2105-5-147)
Supplement: Additional File 2 — The original results of the above study (non-essential files are deleted to keep the file size under the limit set by BMC bioinformatics). [file 1471-2105-5-147-S2.bz2 › chilibotAdditionalFile2/dip40/209ID8846021E709/html/ITGA2_ITGB1.html]

 


 **ITGA2** and **ITGB1** 
  
Found 69 abstracts in PubMed, retrieved 40.  
 

 What does Google say? 
 PDF only 
| .edu only 

---

**Interactive relationship** (e.g. stimulation, inhibition, etc)

**Neutral relationship**- The human integrin very late antigen VLA 2 CD49b  [ **ITGA2** ]  CD29  [ **ITGB1** ]  mediates interactions with collagen and is the receptor for echovirus 1.  Ref: 9353313 J Biol Chem, 1997

**Non-interactive relationship** (e.g. studied together, co-existance, homology, etc.)

- CONCLUSIONS In vivo selection and flow cytometric analysis revealed that Lea, sLea, CD49b  [ **ITGA2** ] , CD49c, and CD29  [ **ITGB1** ]  appear to be involved in the increase of metastatic activity.  Ref: 9733622 J Surg Res, 1998
- In the original line KM12SM, sLea, E cadherin, CD49b  [ **ITGA2** ] , CD49c, or CD29  [ **ITGB1** ]  were positive in more than 40% of the cells.  Ref: 9733622 J Surg Res, 1998
- As model system, we used living human umbilical vein endothelial cells HUVEC adhering to laminin coated surfaces and investigated the distribution of the alpha2beta1 CD29  [ **ITGB1** ]  CD49b  [ **ITGA2** ]  integrin at the contact zone of these cells.  Ref: 12521136 Cell Commun Adhes< MedlineTA>Cell Commun Adhes, 1998
- Data obtained are presented in concurrence with Clusters of Thymic Epithelial Staining CTES classification for various monoclonal antibodies recognizing CD antigens CD1, CD1a, CD6, CD9, CD14, CD16, CD29  [ **ITGB1** ] , CD30, CD32, CD44, CD45RB, CD47, CD48, CD49a, CD49b  [ **ITGA2** ] , CD49c, CD49d, CD49e, CD49f, CD51, CD53, CD54, CD56, CD57, CD63, CD85, CD95, CD98, CD102, CD103, CD106, CD109, CD146, CD147, CD148, CD151, CD152, CD158a, CD158b, CD164, CD165, CD166 and for monoclonal antibodies 1B1 G7, A4, BD46, BLTZ, HP1C5, IND.64, M72, WU947 whose specifities are not yet defined.  Ref: 12725440 Ann Anat, 2003
- We found that T h 1 cells derived from ovalbumin OVA specific TCR transgenic DO11.10 mice showed significantly higher levels of VLA 2 CD49b  [ **ITGA2** ]  CD29  [ **ITGB1** ]  expression than T h 2 cells.  Ref: 12750354 Int Immunol, 2003
- OBJECTIVE We sought to determine whether expression of CD51 vitronectin and fibronectin receptor, CD54 intercellular adhesion molecule 1, HLA DR activation marker, CD29  [ **ITGB1** ]  beta1 integrin, CD49b  [ **ITGA2** ]  collagen receptor  [ **ITGA2** ] , and CD11b complement receptor exhibit a circadian rhythm in asthma.  Ref: 9768576 J Allergy Clin Immunol, 1998
- Flow cytometry of the cell surface antigen expression indicated that CD49b  [ **ITGA2** ] , CD29  [ **ITGB1** ] , carcinoembryonic antigen CEA, sialyl Lewis a sLea, and CD49c were positive in more than 70% of the cells.  Ref: 10853016 Int J Oncol, 2000
- Localization of integrin alpha2  [ **ITGA2** ]  and integrin beta1  [ **ITGB1** ]  in human stellate cells was examined by immunoelectron microscopy.  Ref: 9566533 Anat Rec, 1998
- Immunostaining was performed with a mouse monoclonal anti human integrin alpha2  [ **ITGA2** ]  or integrin beta1  [ **ITGB1** ]  antibody and goat anti mouse IgG coupled with 10 nm immunogold.  Ref: 9566533 Anat Rec, 1998
- Integrin alpha2  [ **ITGA2** ]  and integrin beta1  [ **ITGB1** ]  were detected on the surfaces of cell bodies, cellular processes, and microprojections.  Ref: 9566533 Anat Rec, 1998
- Expression of the CD29  [ **ITGB1** ] , CD49b  [ **ITGA2** ]  and CD31 adhesion molecules on the platelet surface was unaffected by storage in Diatube H.  Ref: 7668222 Am J Hematol, 1995
- TEC were treated with TCDD at concentrations of 0.00.0..0 or 10.0 nM or with 100 nM PCB 126,, 5 pentachlorobiphenyl for 3 days, and were then analysed by flow cytometry for expression of surface antigens using monoclonal antibodies against Hassall s bodies TE 8, TE 16 or against surface structures such as CD29  [ **ITGB1** ] , CD49b  [ **ITGA2** ] , CD49e, CD49f, CD51, CD54, CD58, CD61 and CD106.  Ref: 12799776 Arch Toxicol, 2003
- This platelet aggregation activity was dose dependently inhibited by specific GPIbalpha antibodies, but not by those antibodies against platelet GPIa  [ **ITGA2** ] , GPIIa  [ **ITGB1** ] , GPIIb and GPIIIa.  Ref: 12782089 Toxicon< MedlineTA>Toxicon, 2003
- Anti CD9, CD26, CD29  [ **ITGB1** ] , CD31, CD36, CD44, CD49a, CD49b  [ **ITGA2** ] , CD49c, CD49d, CD49e, CD49f, CD54, CD55, CD56, CD62E &.  Ref: 10235320 J Pediatr Surg, 1999
- HTSC express CD54, CD49a, CD49b  [ **ITGA2** ] , CD49c, CD49e, CD49f, CD29  [ **ITGB1** ] , CD51, CD44 and produce vinculin, beta tubulin, alpha actin, vimentin, fibronectin, laminin and collagen types I, III and IV.  Ref: 8566062 Eur J Immunol, 1996
- Other molecules expressed by the cultured stromal cells include CD29  [ **ITGB1** ] , CD49a, CD49b  [ **ITGA2** ] , CD49d, CD49f, CD51 61, CD54, CD55, CD56, CD58, CD59, CD61, CD71, CD79b, CD81, CD82, CD90, CD95, CD107a, CD130, and CD147.  Ref: 10951495 Prostate, 2000
- Flow cytometric analyses were carried out to detect expression of antigens Lewis a Lea, Lewis x Lex, sialyl Lewis a sLea, sialyl Lewis x sLex, E cadherin, CD44v6, integrin alpha2  [ **ITGA2** ]  CD49b  [ **ITGA2** ] , integrin alpha3 CD49c, integrin alpha4 CD49d, integrin alpha5 CD49e, and integrin beta1  [ **ITGB1** ]  CD29  [ **ITGB1** ] .  Ref: 9733622 J Surg Res, 1998
- We confirmed previous observations that human bronchial epithelial cells constitutively express CD29  [ **ITGB1** ] , CD44, CD49a, CD49b  [ **ITGA2** ] , CD49c, CD49d, CD49e, CD49f, CD51, CD54, CD61, and HLA class 1.  Ref: 9374108 Am J Respir Cell Mol Biol, 1997
- At 14 weeks of culture, more mature CHMCs expressed CD11b, CD11c, CD29  [ **ITGB1** ] , CD49b  [ **ITGA2** ] , CD49c, CD49d, CD49e, CD51, CD61, and CD54 and weakly expressed CD18 and CD11a.  Ref: 11174197 J Allergy Clin Immunol, 2001
- The expression of adhesion structures on mesothelial cells such as CD44, the beta integrin chain CD29  [ **ITGB1** ] , the beta 3 integrin chain CD61 and alpha chains CD49 alpha, CD49b  [ **ITGA2** ] , CD49c, CD49e, and CD51 is described.  Ref: 10649685 Adv Perit Dial, 1998
- In an earlier study we had demonstrated that oncogenic Ki ras4BVal 12, but not oncogenic Ha rasVal 12, blocks the apicobasal polarization of colon epithelial cells by preventing normal glycosylation of the integrin beta1  [ **ITGB1** ]  chain of the collagen receptor  [ **ITGA2** ] .  Ref: 9346938 J Biol Chem, 1997
